# Psychotherapy for Chronic Dizziness: A Scoping Review

**DOI:** 10.7759/cureus.89726

**Published:** 2025-08-10

**Authors:** Shogo Saito, Marina Sato, Fumiyuki Goto

**Affiliations:** 1 Otolaryngology, Goto Otolaryngology Clinic, Machida, JPN; 2 Psychiatry, Umayabashi Hospital, Maebashi, JPN; 3 Otolaryngology, Tokai University School of Medicine, Isehara, JPN

**Keywords:** chronic dizziness, functional dizziness, meniere's disease, persistent postural-perceptual dizziness, psychotherapy, scoping review

## Abstract

Chronic dizziness is a frequent condition that substantially impairs quality of life (QoL), making psychotherapy a crucial intervention alongside physical therapies. However, the overall landscape of evidence regarding its application to diverse dizziness disorders remains unclear. This scoping review aimed to comprehensively map the existing research on this topic, providing an overview of its utilization, intervention methods, and evaluation measures to identify research gaps. Following the Preferred Reporting Items for Systematic reviews and Meta-Analyses extension for Scoping Reviews (PRISMA-ScR) guidelines, we searched PubMed and CiNii Research for peer-reviewed articles published in English or Japanese. Eligible studies were those describing a psychotherapeutic intervention for patients with chronic dizziness lasting three months or longer, published up to September 30, 2023. A total of 644 records were screened, with 23 studies ultimately included for analysis.

Our results indicate that the current evidence base is largely developmental. The most common study design was the case study (10/23 studies, 43.5%), with a median of seven participants. The median age of participants was 45.2 years, indicating a primary focus on adult populations. The predominant psychotherapeutic technique was cognitive behavioral therapy (16/23 studies, 69.6%), typically delivered individually, with a median of 6.5 sessions. While evaluations were often multifaceted, assessing domains such as dizziness symptoms, psychological state, QoL, and physical function, the outcome measures used were highly diverse and not standardized. The Dizziness Handicap Inventory (DHI) and the Hospital Anxiety and Depression Scale (HADS) were the most frequently used instruments. Although the included studies collectively suggest that psychotherapy is a promising intervention for improving symptoms of chronic dizziness, this review highlights several key limitations of the existing evidence base: (a) it consists primarily of small-scale case studies, with a lack of high-quality evidence; (b) the research focuses on adult populations, with a scarcity of studies targeting children or adolescents; (c) while group formats and combination therapies show potential, the research is too limited to draw firm conclusions; and (d) the outcome measures used are highly diverse and not standardized. Therefore, to advance the field, future research must prioritize conducting high-quality intervention studies, particularly large-scale RCTs, that utilize standardized outcome measures.

## Introduction and background

Dizziness is a common physical symptom, with a reported lifetime prevalence of 17-30% [1]. It frequently becomes a chronic condition, persisting for long periods in many cases [2], and spontaneous remission is considered rare in untreated patients [3]. Furthermore, chronic dizziness significantly impairs quality of life (QoL) by causing psychological distress and activity restriction [4]. In addition, psychiatric symptoms such as anxiety and depression have been identified as contributing factors to its chronicity [5]. Indeed, a community-based study reported a significantly higher prevalence of anxiety and avoidance behavior in individuals with dizziness (46%) compared to those without (13.3%) [2]. This interplay between dizziness and psychiatric symptoms is thought to create a vicious cycle that perpetuates the condition, making chronic dizziness a serious condition that substantially impacts individuals' daily lives. Therefore, in addition to physical interventions, psychotherapy is crucial for improving QoL and alleviating psychological symptoms in patients with chronic dizziness.

A meta-analysis by Schmid et al., a seminal study in this field, provided important early evidence by demonstrating a moderate effect size for psychotherapy in treating chronic dizziness [6]. However, the interpretation and generalizability of these findings are subject to several key considerations. As the original authors noted, their analysis was based on only three randomized controlled trials (RCTs) [6]. Furthermore, the review predates the establishment of the diagnostic criteria for persistent postural-perceptual dizziness (PPPD) [7], a condition now recognized as a major and distinct entity within the spectrum of chronic dizziness.

PPPD is a functional disorder characterized by persistent non-rotational dizziness lasting for three months or more. It has a high prevalence, reportedly accounting for approximately 25% of dizziness diagnoses in adults [8]. PPPD is classified as either secondary, following a somatic trigger such as a vestibular disorder, or primary, in the absence of such a trigger. According to a registry-based study, 55% of cases are primary, and notably, these patients with primary PPPD have a significantly higher prevalence of psychiatric comorbidities, such as depressive and anxiety disorders, compared to those with secondary PPPD [9]. Given its high prevalence and strong association with psychological factors, the absence of PPPD as a diagnostic entity in the 2011 meta-analysis [6], combined with the small number of studies included, significantly constrains the generalizability of its findings to the diverse spectrum of chronic dizziness seen today.

Since the 2011 report by Schmid et al. [6], there has been a lack of comprehensive reviews summarizing the current evidence for psychotherapy in chronic dizziness, particularly one that accounts for these subsequent developments in diagnostic concepts. This highlights a clear need to map the current landscape of applications, characterize the existing evidence, and identify research gaps. Therefore, a scoping review methodology was chosen for this study. The primary aim of a scoping review is to map the overall landscape of existing knowledge and identify research gaps, which directly aligns with the needs of this field [10]. Accordingly, the objective of this scoping review is to examine the existing research on psychotherapy for chronic dizziness lasting three months or longer, providing an overview of its utilization, intervention methods, and evaluation strategies, and to clarify the agenda for future research.

Portions of this work were previously presented as a poster at the 83rd Annual Meeting of the Japan Society for Equilibrium Research on November 14, 2024, in Aichi, Japan.

## Review

Methods

This study was conducted as a scoping review and reported in accordance with the Preferred Reporting Items for Systematic reviews and Meta-Analyses extension for Scoping Reviews (PRISMA-ScR) guidelines [11]. A review protocol was not registered for this study, as this is not a mandatory requirement for scoping reviews.

Review Questions and PCC Framework

This review was guided by the following questions: (a) How is psychotherapy utilized for chronic dizziness? (b) What types of psychotherapy are used for chronic dizziness? (c) How are the effects of psychotherapy and the improvement of chronic dizziness evaluated?

The Population, Concept, Context (PCC) framework was used to structure these questions and the eligibility criteria as follows: (i) Population: Patients with chronic dizziness lasting for three months or longer; (ii) Concept: Psychotherapeutic interventions, including those delivered alone or in combination with other treatments; (iii) Context: No limitations on the age, country, or geographical region of the subjects.

Eligibility Criteria

Studies were included if they met the following criteria: (a) targeted patients with chronic dizziness lasting for three months or longer; (b) included a psychotherapeutic intervention; (c) were peer-reviewed articles; and (d) were written in English or Japanese. Review articles were excluded.

Information Sources and Search Strategy

We searched the PubMed and CiNii Research databases for English and Japanese articles, respectively. The search period was not limited by a start year and concluded with studies published up to September 30, 2023 (final search conducted on December 4, 2023). To ensure comprehensive coverage, we also hand-searched the reference lists of included studies and relevant review articles. The search strategy was constructed around two key concepts: the condition (i.e., dizziness) and the intervention (i.e., psychotherapy). The search formula combined terms for the dizziness concept (e.g., "dizziness") with terms for the psychotherapy concept (e.g., "counseling," "psychotherapy") using the 'AND' operator. The full electronic search strategy for PubMed is detailed in Appendix A.

Study Selection and Data Extraction

A standardized data charting form was developed to extract key information from each included study. Two researchers (SS and MS) independently extracted data from all studies. For articles published in Japanese, the data were first extracted in their original language. After reaching a consensus on the extracted Japanese data, the first author (SS), a native Japanese speaker, translated the information into English. The accuracy and consistency of this translation were then verified by the second author (MS) against the original Japanese articles.

Any disagreements that arose during the data extraction process, including those during translation verification, were resolved through discussion between the first two authors, with arbitration by a third researcher (FG) if consensus could not be reached.

The following key data were extracted from each study: Author, Country, Study design, Diagnosis, Number of participants, Age, Psychotherapeutic techniques, Number of sessions, and Outcome measures.

Data Synthesis

We calculated descriptive statistics (i.e., median, mean, and range) to summarize participant age and the number of intervention sessions from the included studies. For this quantitative synthesis, the following criteria were applied: (a) studies providing non-specific data, such as age reported only by decade (e.g., "30s") or an indeterminate number of sessions, were excluded; and (b) when a single study provided multiple data points for a variable (e.g., mean ages for different treatment groups or multiple session counts within one report), each data point was treated as an independent observation for aggregation.

Quality Assessment

Consistent with the primary aim of a scoping review, which is to map the breadth of evidence regardless of its methodological quality, a formal critical appraisal of the included sources of evidence was not performed. This approach aligns with the PRISMA-ScR guidelines, which consider this step optional. Given the diversity of the evidence base, which included various study designs from RCTs to case studies, applying a single, standardized appraisal tool was deemed inappropriate for this review.

Results

**Figure 1:**
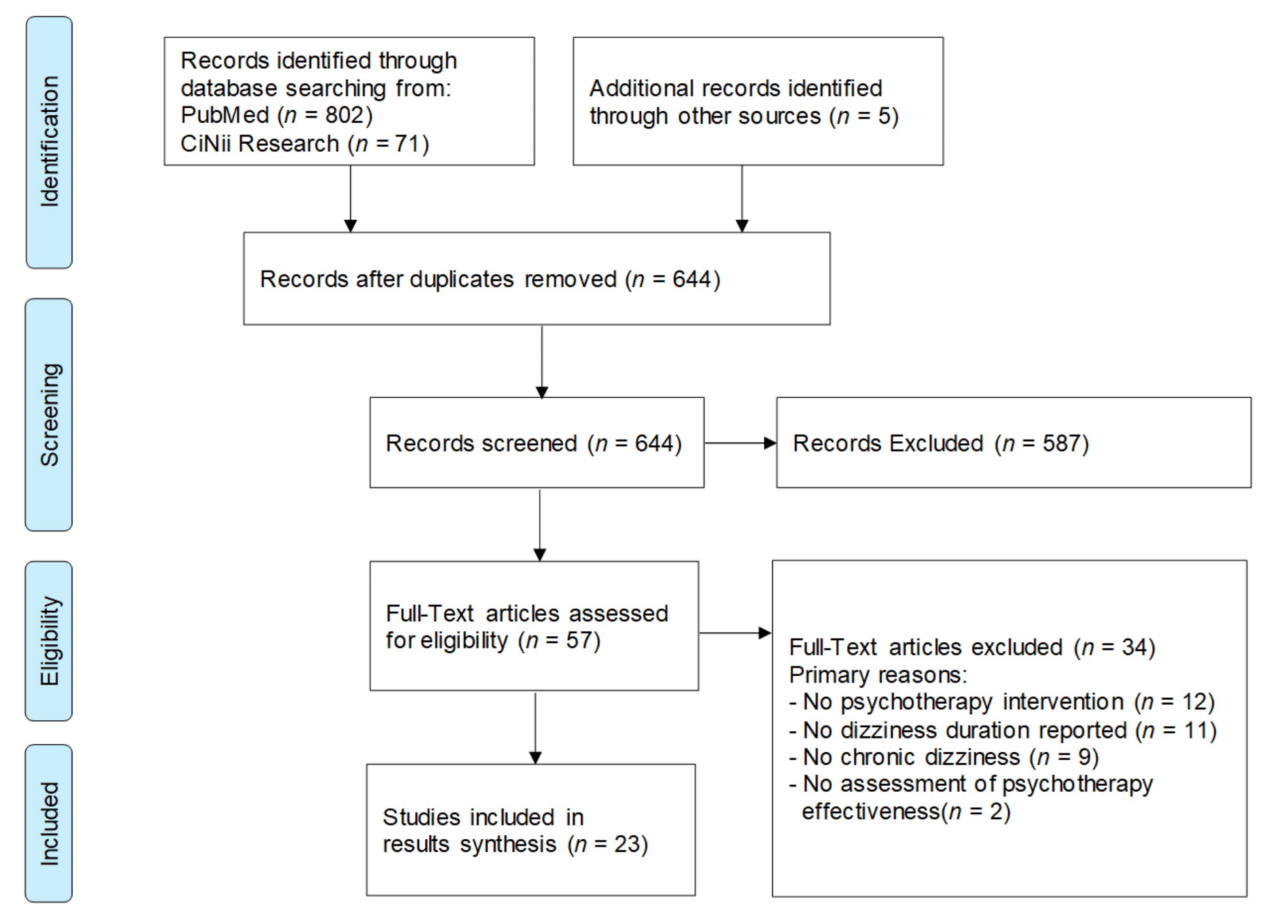
PRISMA-ScR flow diagram of the study selection process PRISMA-ScR: Preferred Reporting Items for Systematic reviews and Meta-Analyses extension for Scoping Reviews

A total of 873 studies were identified through database searching, and five studies were identified through hand-searching, resulting in 878 studies overall. After removing duplicates, 644 studies remained. Following title and abstract screening, 587 studies were excluded, leaving 57 studies for full-text screening. Ultimately, 23 studies met the eligibility criteria (Figure 1).

Characteristics of Included Studies

An overview of the included studies is presented in Table 1. The 23 studies were published between 1991 and 2023, with the distribution by decade as follows: four studies (17.4%) in the 1990s, six (26.1%) in the 2000s, five (21.7%) in the 2010s, and eight (34.8%) in the 2020s. Regarding the country of study, 14 studies (60.9%) were conducted in Japan, followed by two (8.7%) in the United Kingdom, and one each (4.3%) in Germany, Sweden, Norway, Switzerland, the United States, Australia, and China. Of the 23 studies, 14 (60.9%) were published in English and nine (39.1%) in Japanese; all of the Japanese-language studies were conducted in Japan.

**Table 1:** Summary of characteristics of the 23 included studies AAO-HNS, American Academy of Otolaryngology–Head and Neck Surgery; AAQ-II, Acceptance and Action Questionnaire–II; ACT, Acceptance and Commitment Therapy; AT, autogenic training; BCI, Balance Control Index; BDI, Beck Depression Inventory; BF, biofeedback; BSI, Brief Symptom Inventory; BPPV, benign paroxysmal positional vertigo; BSQ, Body Sensation Questionnaire; CBS, Cognitive Bias Scale; CBT, Cognitive behavioral Therapy; CMI, Cornell Medical Index; CSD, chronic subjective dizziness; DASS-21, Depression Anxiety Stress Scales-21; DHI, Dizziness Handicap Inventory; DO, dizziness only; DSI, Dizziness Symptoms Inventory; EMG, electromyography; EQ-5D-3L, EuroQol 5-dimensions 3-level; EQ-5D-5L, EuroQol 5-dimensions 5-level; FFMQ, Five Facet Mindfulness Questionnaire; GAF, Global Assessment Functioning Scale; HADS, Hospital Anxiety and Depression Scale; HARS, Hamilton Anxiety Rating Scale; HDRS, Hamilton Depression Rating Scale; HmDizz, perceived dizziness before and after head movements; mCTSIB, Modified Clinical Test for Sensory Interaction and Balance; MI-A, Mobility Inventory of Agoraphobia-Alone; MD, Meniere's disease; MPI, Maudsley Personality Inventory; NPQ, Niigata PPPD Questionnaire; PPPD, persistent postural perceptual dizziness; PPV, phobic postural vertigo; QBD, quantified balance disorders; RCT, randomized controlled trial; SDS, Self-rating Depression Scale; SHC, subjective health complaints; STAI, State-Trait Anxiety Inventory; STAI-t, State-Trait Anxiety Inventory-trait version; SRQ-D, Self-Rating Questionnaire for Depression; SUD, subjective units of distress; TEG, Tokyo University Egogram; VAS, Visual Analog Scale; VSS, Vertigo Symptom Scale; VSS-sf, Vertigo Symptom Scale-short form; VVAS, Visual Vertigo Analogue Scale; %TSI, percentage of time symptoms interfere with life

Author	Country	Study design	Diagnosis	Number of participants	Age	Techniques	Sessions	Outcomes
Axer et al., 2020 [12]	Germany	Observational study	PPPD; Chronic dizziness	657 (PPPD: 305; Chronic dizziness: 352)	PPPD (50.4±14.0); Chronic dizziness (63.9±13.2)	Multimodal treatment (day care program)	5 days/week, 7 hours of therapy daily	HADS; VAS (vertigo/dizziness intensity, distress); VSS
Goto et al., 2005 [13]	Japan	Case study	Fibromyalgia	1	39	AT; Breathing technique; CBT	18	not reported
Goto et al., 2008 [14]	Japan	Case study	MD	1	51	AT; CBT	6	not reported
Goto et al., 2008 [15]	Japan	Case study	PPV	1	37	AT	6	not reported
Goto et al., 2011 [16]	Japan	Observational study	MD	6	56.7±17.8	AT	4-19	6-point functional level scale based on AAO-HNS guidelines
Herdman et al., 2022 [17]	United Kingdom	RCT	PPPD	40 (Treatment: 20; Control: 20)	Treatment (44.6±17.0); Control (44.3±17.4)	CBT	6	EQ-5D-5L; DHI; VVAS; %TSI
Johansson et al., 2001 [18]	Sweden	RCT	Chronic dizziness	22 (Treatment: 11; Control: 11)	71.8±5.2	Group CBT	5	BDI; DHI; Minimum time to perform 6 basic exercises; Sharpened Romberg test; STAI-t; VSS-sf; 10m walk speed
Kang et al., 2023 [19]	Japan	Case study	PPPD	2	40s	CBT	6	DHI; HADS; NPQ
Kanzaki et al., 2001 [20]	Japan	Case study	MD	6	34-58	AT (individual, group); BF; CBT; Progressive muscle relaxation; Psychoanalysis	not reported	Pure-tone audiometry
Kojima et al., 2023 [21]	Japan	Before-after study	PPPD	13	45.5±13.6	CBT	8	CBS; DHI; HADS; STAI
Kondo, 2015 [22]	Japan	Before-after study	Chronic dizziness	22	48.7±12.6	Group CBT	4-5	DHI
Kristiansen et al., 2019 [23]	Norway	Before-after study	Chronic dizziness	7	38±8	Group CBT	8	BSQ; Clinical dynamic visual acuity; DHI; Dual task walking test; EQ-5D-5L; Global physiotherapy examination; Grip strength; HADS; HmDizz; mCTSIB; MI-A; Panic attack scale-modified version; Sharpened Romberg test; SHC; Timed Up-and-Go; VSS-sf; 6m walk speed
Kuwabara et al., 2020 [24]	Japan	Before-after study	PPPD	27	45.2±9.9	Group ACT	6	AAQ-Ⅱ; DHI; FFMQ; HADS; VSS-sf
Nakai et al., 2008 [25]	Japan	Case study	MD	1	30s	CBT	25	CMI; GAF; MPI; SDS; SUD (dizziness, hyperventilation, tinnitus); TEG
Nakayama et al., 1998 [26]	Japan	Before-after study	Chronic dizziness	26	47.4±15.4	Group AT; Group psychotherapy	1	CMI; Evaluation of satisfaction with treatment; SRQ-D; Stabilometry
Saito and Goto, 2022 [27]	Japan	Case study	PPPD	2	20s-30s	Morita therapy	5-12	DHI; HADS
Sakai et al., 1992 [28]	Japan	Case study	MD	1	34	Transactional analysis	not reported	Pure-tone audiometry
Sama et al., 1995 [29]	United States	Case study	Hyperventilation	3	26-45	Behavior therapy; Relaxation techniques	not reported	not reported
Schmid et al., 2018 [30]	Switzerland	Before-after study	Chronic dizziness	32 (DO: 16; QBD: 16)	DO (60.6±8.3); QBD (44.8±12.1)	CBT	8	BCI; BSI; DHI; EQ-5D-3L
Shutty et al., 1991 [31]	United States	Case study	BPPV	1	26	Behavior therapy	9	Frequency of dizzy spells; Frequency and intensity ratings of headaches; Physical activities; Severity (dizziness, anxiety, muscle tension; 1-10 scale); Surface EMG
Toshishige et al., 2020 [32]	Japan	Before-after study	CSD	37	46.1±13.3	Group CBT	5-6	DHI
Waterston et al., 2021 [33]	Australia	Observational study	PPPD	150	46	CBT	up to 6 (mean 4.8)	Avoidance and safety behaviors; DASS-21; DHI; DSI
Yu et al., 2018 [34]	China	RCT	PPPD	91 (Treatment: 46; Control: 45)	Treatment (42.7±9.8); Control (42.2±9.6)	CBT	16	Adverse events; DHI; HARS; HDRS; Sertraline dose

The study designs included case studies (10 studies, 43.5%), before-after studies (seven studies, 30.4%), observational studies (three studies, 13.0%), and RCTs (three studies, 13.0%). A majority of the studies were small-scale; for instance, all 10 case studies involved 10 or fewer participants, with six being single-participant studies and four including two to six participants. In contrast, two observational studies (8.7%) reported sample sizes of over 100 participants.

Participant Characteristics

A total of 1150 participants were included across the 23 studies. The sample size per study ranged from 1 to 657, with a median of 7 and a mean of 50.

Information on participant age was reported in all 23 studies, though the reporting format varied. For the quantitative synthesis, three studies that only reported age by decade were excluded, leaving numerical data from 20 studies. These 20 studies yielded a total of 31 data points for aggregation. Based on these 31 data points, the median age was 45.2 years, and the mean age was 46.1 years (range: 26 to 71.8 years). While one study (4.3%) specifically targeted older adults (mean age >65 years) [18], no studies focusing on children or adolescents were identified.

Regarding the participants' diagnoses, the most frequently studied condition was PPPD, including related disorders such as phobic postural vertigo (PPV) and chronic subjective dizziness (CSD), which accounted for 10 studies (43.5%). This was followed by Meniere's disease (five studies, 21.7%) and non-specific chronic dizziness (five studies, 21.7%). Fibromyalgia, hyperventilation, and benign paroxysmal positional vertigo (BPPV) were each reported in one study (4.3%).

Characteristics of Interventions

The psychotherapeutic techniques, delivery formats, and intervention durations identified in this review were diverse. The most common technique was cognitive behavioral therapy (CBT) (16 studies, 69.6%), followed by autogenic training (AT) (seven studies, 30.4%). Regarding the delivery format, 16 studies (69.6%) implemented psychotherapy in an individual-only format, six (26.1%) in a group-only format, and one (4.3%) used a combination of both. Combination with other treatments was also observed in several studies; five studies (21.7%) combined psychotherapy with vestibular rehabilitation (VR), and one study (4.3%) combined it with a selective serotonin reuptake inhibitor (SSRI).

Information regarding the number of intervention sessions was available in 20 of the 23 included studies (87.0%). After excluding four studies for which a specific number of sessions could not be determined, a total of 22 data points from the remaining 16 studies were used for the analysis. The median number of sessions was 6.5, and the mean was 8.32 (range: 1-25).

Despite this diversity in techniques, formats, and durations, an overarching finding from this review was that psychotherapy consistently contributed to improvements in dizziness and associated symptoms across the included studies. To explore this finding in greater detail, the specific outcomes for the different clinical conditions identified in this review are now presented.

Persistent Postural-Perceptual Dizziness

Ten studies targeted PPPD, including two RCTs. In the study by Yu et al., the group receiving adjunctive CBT showed significantly greater improvements in the Dizziness Handicap Inventory (DHI), the Hamilton Anxiety Rating Scale (HARS), and the Hamilton Depression Rating Scale (HDRS) compared to the SSRI monotherapy group; the SSRI dosage and the incidence of adverse events were also significantly lower [34]. In a feasibility RCT by Herdman et al., where a CBT-informed VR intervention was compared to standard VR, 60.0% of participants (12 of 20) in the CBT combination group achieved a reliable improvement on the DHI (a reduction of >18 points), compared to 35.0% (seven of 20) in the control group [17].

In observational studies, Waterston et al. reported that CBT led to significant improvements in the DHI, the Dizziness Symptoms Inventory (DSI), the Depression Anxiety Stress Scales-21 (DASS-21), and Avoidance and safety behaviors with effects maintained at the six-month follow-up [33]. Several pre-post comparison studies have also been conducted. Toshishige et al. showed that group CBT significantly improved the DHI up to the six-month follow-up [32]. They also found that the presence of a comorbid anxiety disorder was a predictor of this improvement. In contrast, Kojima et al. reported that while CBT also improved some measures, such as the DHI physical subscale and the State-Trait Anxiety Inventory (STAI), there were no significant changes in the total DHI or the Hospital Anxiety and Depression Scale (HADS) [21]. Regarding other therapies, Kuwabara et al. reported that with group acceptance and commitment therapy (ACT), 40.7% (11 of 27) of patients achieved significant remission on the DHI, which was maintained for up to six months [24].

At the case report level, various psychotherapies, including CBT, AT, and Morita therapy, were reported to result in partial or complete improvement in dizziness and symptoms of anxiety and depression [15,19,27].

Meniere's Disease

Five studies targeting Meniere's disease primarily investigated AT. A small-scale observational study showed that AT led to symptom improvement in 83.3% (five of six) of patients, based on the American Academy of Otolaryngology-Head and Neck Surgery (AAO-HNS) Functional Level Scale [16]. Case reports have described the cessation of vertigo attacks and improvements in hearing and tinnitus following various psychotherapies, including AT, CBT, and Transactional Analysis [14,20,25,28].

Non-Specific Chronic Dizziness

Six studies targeted non-specific chronic dizziness. The only RCT in this category, by Johansson et al., compared a combined CBT and VR intervention against a waitlist control group in older adults [18]. The results showed that the treatment group had significantly greater improvements in the DHI and walking performance compared to the control group, but no significant differences were found on the STAI trait anxiety subscale or the Beck Depression Inventory (BDI).

Observational studies have shown mixed findings. A study on multidisciplinary treatment by Axer et al. found significant improvements in the VSS and the HADS Anxiety subscale, but no improvement in the HADS depression subscale was observed [12]. In a study investigating a combined group CBT and VR intervention, Schmid et al. reported that the outcome differed based on the patients' physical status [30]. While the DHI significantly improved in the patient group without objective balance impairment, only balance function improved in the group with balance impairment, with no change in the DHI. Furthermore, their study found a strong, significant correlation between phobic anxiety and the DHI in both patient groups.

In pre-post comparison studies, Kondo reported that 45.5% (10 of 22) of patients achieved significant remission on the DHI up to the 6-month follow-up with group CBT [22]. In a feasibility study of a group intervention combining CBT and VR, Kristiansen et al. found that 40.0% (two of five) of patients on the DHI and 66.7% (two of three) on gait speed demonstrated improvements exceeding the minimal clinically important difference [23]. Nakayama et al. reported improvements in the Cornell Medical Index (CMI), the Self-Rating Questionnaire for Depression (SRQ-D), and posturography with eyes closed following group psychotherapy [26].

Other Disorders

For several other disorders, only single case reports or small case series met the eligibility criteria. A case report on fibromyalgia reported improvements in pain and dizziness with psychotherapy that included CBT and AT [13]. In a case series on hyperventilation syndrome, two of four cases showed improvement in dizziness with behavioral therapy and relaxation techniques [29]. Additionally, a case report on BPPV reported a 90% reduction in vertigo attacks after nine weeks of behavioral therapy [31].

Outcome Measures

Of the 23 included studies, 19 (82.6%) provided clear information on outcome measures, whereas the remaining four (17.4%) did not provide sufficient detail. Dizziness symptoms were evaluated using subjective or objective measures in all 19 of these studies. Unless otherwise specified, the usage counts and percentages for each outcome measure presented in this section were calculated using the total number of included studies (N=23) as the denominator.

A wide variety of measures were used for outcome evaluation (see Table 1 for details). For the assessment of dizziness symptoms, the DHI was the most frequently used measure, reported in 11 studies (47.8%). Other self-report questionnaires included the Vertigo Symptom Scale - short form (VSS-sf) in three studies (13.0%), the Visual Vertigo Analogue Scale (VVAS) in one study (4.3%), and the Niigata PPPD Questionnaire (NPQ) in one study (4.3%). In addition, objective measures were utilized in seven studies (30.4%), such as posturography, Pure-Tone Audiometry, and gait tests (e.g., 10 m walk speed). Overall, regarding the evaluation of dizziness symptoms, subjective measures were used in 15 studies (65.2%) and objective measures in seven studies (30.4%), while three studies (13.0%) employed both.

Of the 23 included studies, 13 (56.5%) reported using at least one measure of psychological symptoms. The most frequently used instrument was the HADS, which assesses both anxiety and depression, and was utilized in six studies (26.1%). Other scales assessing general psychological distress or a wide range of psychological symptoms included CMI in two studies (8.7%), the DASS-21 in one study (4.3%), and the Brief Symptom Inventory (BSI) in one study (4.3%). Measures specific to anxiety included the STAI (two studies, 8.7%) and the HARS (one study, 4.3%), while those specific to depression included the HDRS and the BDI, each used in one study (4.3%).

In addition, measures of health-related QoL were utilized. The EuroQol 5-dimensions (EQ-5D) was used in a total of three studies (13.0%), comprising the 5L version in two studies and the 3L version in one study. Other instruments assessing broader functional or psychological aspects were also identified, including the Acceptance and Action Questionnaire-II (AAQ-II), which was used in one study (4.3%).

Discussion

The objective of this study was to provide an overview of the current status of psychotherapy for chronic dizziness by examining how it is utilized, what types of interventions are applied, and how its effects are evaluated. This scoping review identified 23 eligible studies. A key finding is that, despite variations in research design and intervention techniques, the included studies consistently reported that psychotherapy contributed to improvements in dizziness and associated symptoms. However, the limited number of studies, the small-scale nature of the research (median participants: 7), and the predominance of case studies (approx. 40%) also suggest that the evidence base in this field is still in a developmental stage.

Utilization Context of Psychotherapy

The focus of research in this field has notably shifted in recent years, with a growing concentration on functional dizziness, particularly PPPD. This likely reflects a growing interest in psychotherapy for this condition as a new therapeutic target, in line with the establishment and dissemination of this disease concept [35]. On the other hand, the fact that psychotherapy has also been trialed for conditions such as Meniere's disease and non-specific chronic dizziness suggests its potential applicability to patients with diverse clinical backgrounds. Therefore, further investigation into the role and efficacy of psychotherapy tailored to these specific disease entities is warranted.

The age composition of the participants was centered on adult populations. While a few studies targeted older adults, such as the one by Johansson et al. [18], no studies focusing on children or adolescents were identified within the scope of this review. This represents a significant research gap, as dizziness in children is known to have different characteristics from that in adults regarding its causes and prevalence [36]. Consequently, future research should prioritize assessing the psychosocial needs of these under-researched age groups, as well as developing and validating age-appropriate intervention methods.

Predominant Therapeutic Approaches and Evidence Gaps

The current evidence landscape for psychotherapy in chronic dizziness is characterized by the predominance of CBT, as evidenced by its use in 16 of the 23 included studies, three of which were RCTs. Indeed, a recent meta-analysis on PPPD showed that adding CBT to conventional treatments significantly improves not only dizziness handicap but also psychological symptoms such as anxiety and depression [37]. This meta-analysis provides quantitative support for the augmenting impact of CBT, at least within the key patient population of PPPD. While the applicability of these findings to the broader spectrum of chronic dizziness warrants further investigation, it nonetheless highlights that CBT is the most extensively studied and evidence-supported psychotherapeutic approach for this condition to date.

In contrast to the extensive evidence for CBT, other techniques such as AT, ACT, and Morita therapy were supported by limited high-quality evidence, as they were mainly reported in case studies or non-randomized trials. For these emerging techniques, further exploratory studies are needed before their efficacy can be robustly evaluated.

Potential of Group Therapy as a Delivery Format

While individual therapy remains the most frequently studied delivery format, the potential of group therapy represents a significant, underexplored area, particularly given the psychosocial aspects of chronic dizziness such as loneliness and loss of identity [38]. This is supported by meta-analytic evidence in analogous chronic conditions like chronic pain, suggesting its potential value in this population as well [39].

This potential is further supported by evidence from our included studies. Participants in the group therapy feasibility study by Kristiansen et al. reported that peer support and shared experiences were motivating and beneficial [23]. This finding suggests that such group-specific therapeutic factors are an important component of this delivery format, underscoring the need for future research to verify the effectiveness and optimal content of group-based interventions.

Intervention Dose and Duration

While the interventions identified in this review were generally short-term (median: 6.5 sessions), this finding must be interpreted with caution due to the nature of the available evidence. The predominance of descriptive designs, such as case studies with primary purpose of describing phenomena rather than to formally evaluate efficacy, is inherently unsuitable for establishing an optimal dose-response relationship. Therefore, future research using more rigorous study designs, particularly RCTs designed for purposes such as comparative effectiveness, is essential to determine the ideal intervention dose, including the number, frequency, and overall duration of sessions.

Pivotal Role of Combination and Multimodal Approaches

A key theme that emerged from the evidence mapped in this review is the potential of combining psychotherapy with other modalities. Although there are differences in study designs, participants, and outcome measures, several studies suggest that combination therapy may be more effective than psychotherapy alone. For example, a CBT-informed VR intervention [17], the combination of an SSRI and CBT [34], and a multidisciplinary program integrating psychotherapy, VR, and pharmacotherapy [12] have all reported positive outcomes.

The theoretical rationale supporting the effectiveness of such combination therapies can be attributed to the pathological model of PPPD proposed by Popkirov et al. [40]. This model views PPPD as a "cycle of maladaptation" in which physical maladaptation of balance control and psychological-behavioral factors, such as fear and avoidance, form a vicious cycle. It is thought that by having VR address the physical aspects and CBT simultaneously address the psychological aspects, the likelihood of breaking this vicious cycle increases.

This view is also supported by the recent systematic review [41]. They confirmed small to moderate effects across diverse interventions and recommended a multimodal approach integrating psychotherapy, vestibular rehabilitation, and medication support. However, it is important to note that in both our review and theirs, the evidence for these combination therapies is predominantly derived from small-scale studies, many of which lack rigorous comparative designs.

These findings suggest that a promising future direction is tailored, multimodal care that combines interventions according to individual patient characteristics, rather than applying a specific therapy in a one-size-fits-all manner. However, they also highlight that more large-scale, comparative research is essential to establish which therapeutic combinations are most effective for specific patient populations.

Challenges in Outcome Measurement

Across the studies identified in this review, a significant diversity was observed in the outcome measures used. Although the DHI and HADS emerged as the most frequently utilized instruments, the field lacks overall standardization. This heterogeneity poses a significant barrier to comparing results across studies and conducting evidence synthesis.

The reason for this diversity may lie in the multifaceted nature of chronic dizziness itself. While the importance of a multifaceted evaluation, encompassing symptoms, psychological state, QoL, and physical function, is well-recognized, this very need may paradoxically contribute to the diversity of measures. This is reflected in our finding that subjective measures were predominant (used in 15 studies), particularly for assessing key related domains such as psychological state and QoL.

To address the challenge that this complexity in evaluation poses to the aggregation of findings, we strongly recommend the development and active use of a core outcome set (COS). Adopting a COS that includes reliable and valid subjective and objective measures covering these key domains would reduce heterogeneity, facilitate meaningful evidence synthesis, and ultimately advance clinical practice.

Limitations

The significance of this review lies in its comprehensive mapping of the research on psychotherapy for chronic dizziness, which clarifies the current evidence landscape and identifies key research gaps. However, several limitations must be acknowledged. First, in accordance with the scoping review methodology, an assessment of the quality of individual studies was not conducted. Therefore, the validity of the findings from the primary studies has not been evaluated, and the results of this review should be interpreted with caution. Second, the search was restricted to peer-reviewed articles, and therefore, unpublished studies and grey literature were not included. Consequently, the potential for publication bias, wherein studies with positive results are more likely to be published, cannot be entirely ruled out. Third, by limiting our search to English and Japanese literature, we may have omitted relevant studies published in other languages, potentially affecting the generalizability of our findings. The high proportion of studies from Japan identified in this review is likely, in part, a consequence of this linguistic scope, suggesting a limitation in the comprehensiveness of our overview of research trends in other regions.

## Conclusions

This scoping review provided an overview of the current status of psychotherapy for chronic dizziness lasting three months or longer. Although the included studies have methodological limitations, they collectively suggest that psychotherapy may be a promising intervention for improving chronic dizziness symptoms, associated psychological distress, and functional impairment. However, our findings also reveal that the existing evidence base is largely composed of small-scale studies (median participants: 7), with case studies being the predominant research design, and that the outcome measures used are diverse and not standardized.

To advance the field and build a more robust evidence base, the future research agenda should be multifaceted. Priorities include not only conducting high-quality RCTs but also direct comparisons between major therapeutic techniques, investigations into optimal delivery formats and intervention doses, and verification of the role of psychotherapy within multimodal care models. Furthermore, the development and adoption of a COS is also essential to facilitate meaningful evidence synthesis.

## Appendices

PubMed search strategy

The search was conducted on December 4, 2023. The search terms were combined as follows:

Concept 1: Dizziness

("dizziness"[All Fields] OR "vertigo"[All Fields])

AND

Concept 2: Psychotherapy

("counseling"[All Fields] OR "counselling"[All Fields] OR "psychotherapy"[All Fields] OR "psychoeducation"[All Fields] OR "cognitive behavioral therapy"[All Fields] OR "cognitive therapy"[All Fields] OR "behavior therapy"[All Fields])

Filters Applied: Publication Date: up to September 30, 2023
